# Preserving the Activities of Daily Living Independence in Late Life

**DOI:** 10.1093/geroni/igaa057.735

**Published:** 2020-12-16

**Authors:** Pao-feng Tsai, Thomas Jakobs, Reid Landes

**Affiliations:** 1 Auburn University, Auburn, Alabama, United States; 2 InvoTek Inc., Alma, Arkansas, United States; 3 University of Arkansas for Medical Sciences, Little Rock, Arkansas, United States

## Abstract

Levels of Assistance (LoA) is an effective caregiving intervention for maintaining activity of daily living (ADL) independence. It is a structured, almost prescriptive, approach to encourage completing ADLs as independently as an elder’s capabilities permit. With appropriate prompts and assistance during dressing, elders can overcome disability, express retained competencies, and experience success. Simultaneously, caregivers learn to view their functions as maintaining the quality of life of able elders, and they receive reinforcement from elders who are more confident and happier. This study is a continuation of a previous project that created and tested a computer application training program for LoA in nursing homes. We refined the app to include grooming LoA and tested on 10 certified nursing assistant (CNA)/resident dyads at a local nursing home. The pilot results showed, although we did not see consistent improvement in CNA’s dressing LoA, we achieved 10% to 30% improvement in grooming LoA. This indicates that the dressing assistance training is able to transfer to grooming LoA. With only an average of one-hour app training, this improvement is cost effective as compared to training provided by care professionals. Future studies should consider incorporating a culture change strategy to improve CNAs’ intention for assisting elders. In addition, the training program should be offered in the initial hire to achieve maximum effect.

